# What are the clinical symptoms and physical signs for non‐small cell lung cancer before diagnosis is made? A nation‐wide multicenter 10‐year retrospective study in China

**DOI:** 10.1002/cam4.2256

**Published:** 2019-05-31

**Authors:** Pu‐Yuan Xing, Yi‐Xiang Zhu, Le Wang, Zhou‐Guang Hui, Shang‐Mei Liu, Jian‐Song Ren, Ye Zhang, Yan Song, Cheng‐Cheng Liu, Yun‐Chao Huang, Xian‐Zhen Liao, Xiao‐Jing Xing, De‐Bin Wang, Li Yang, Ling‐Bin Du, Yu‐Qin Liu, Yong‐Zhen Zhang, Yun‐Yong Liu, Dong‐Hua Wei, Kai Zhang, Ju‐Fang Shi, You‐Lin Qiao, Wan‐Qing Chen, Jun‐Ling Li, Min Dai, the LuCCRES Group

**Affiliations:** ^1^ National Cancer Center/National Clinical Research Center for Cancer/Cancer Hospital Chinese Academy of Medical Sciences and Peking Union Medical College Beijing China; ^2^ Affiliated Hospital of Guizhou Medical University, Guizhou Province Tumor Hospital Guiyang P.R. China; ^3^ Tumor Hospital of Yunnan Province Kunming P.R. China; ^4^ Hunan Cancer Hospital Changsha P.R. China; ^5^ Liaoning Cancer Hospital & Institute Shenyang P.R. China; ^6^ Anhui Medical University Hefei P.R. China; ^7^ Guangxi Medical University Nanning P.R. China; ^8^ Zhejiang Cancer Hospital Hangzhou P.R. China; ^9^ Gansu Provincial Cancer Hospital Lanzhou, Gansu P.R. China; ^10^ Shanxi Provincial Cancer Hospital Taiyuan P.R. China; ^11^ Anhui Provincial Cancer Hospital Hefei P.R. China

**Keywords:** clinical stage, nonsmall cell lung cancer (NSCLC), pathological type, physical signs, symptoms

## Abstract

**Background:**

Most lung cancer patients are diagnosed after the onset of symptoms. However, whether the symptoms of lung cancer were independently associated with the diagnosis of lung cancer is unknown, especially in the Chinese population.

**Methods:**

We conducted a 10 years (2005‐2014) nationwide multicenter retrospective clinical epidemiology study of lung cancer patients diagnosed in China. As such, this study focused on nonsmall cell lung cancer (NSCLC). We calculated the odds ratios (ORs) for variables associated with the symptoms and physical signs using multivariate unconditional logistic regressions.

**Results:**

A total of 7184 lung cancer patients were surveyed; finally, 6398 NSCLC patients with available information about their symptoms and physical signs were included in this analysis. The most common initial symptom and physical sign was chronic cough (4156, 65.0%), followed by sputum with blood (2110, 33.0%), chest pain (1146, 17.9%), shortness of breath (1090, 17.0%), neck and supraclavicular lymphadenectasis (629, 9.8%), weight loss (529, 8.3%), metastases pain (378, 5.9%), fatigue (307, 4.8%), fever (272, 4.3%), and dyspnea (270, 4.2%). Patients with squamous carcinoma and stage III disease were more likely to present with chronic cough (*P* < 0.0001) and sputum with blood (*P* < 0.0001) than patients with other pathological types and clinical stages, respectively. Metastases pain (*P* < 0.0001) and neck and supraclavicular lymphadenectasis (*P* = 0.0006) were more likely to occur in patients with nonsquamous carcinoma than in patients with other carcinomas. Additionally, patients with stage IV disease had a higher percentage of chest pain, shortness of breath, dyspnea, weight loss, and fatigue than patients with other stages of disease. In multivariable logistic analyses, compared with patients with adenocarcinoma, patients with squamous carcinoma were more likely to experience symptoms (OR = 2.885, 95% confidence interval [CI] 2.477‐3.359) but were less likely to present physical signs (OR = 0.844, 95% CI 0.721‐0.989). The odds of having both symptoms and physical signs were higher in patients with late‐stage disease than in those with early‐stage disease (*P* < 0.0001).

**Conclusions:**

The symptoms and physical signs of lung cancer were associated with the stage and pathological diagnosis of NSCLC. Patients with squamous carcinoma were more likely to develop symptoms, but not signs, than patients with adenocarcinoma. The more advanced the stage at diagnosis, the more likely that symptoms or physical signs are to develop. Further prospective cohort studies are needed to explore these results.

## 1 INTRODUCTION

Lung cancer is the most common cancer and the leading cause of cancer mortality worldwide, accounting for more than 2.0 million newly diagnosed cancer cases and 1.7 million cancer‐related deaths each year.^1^ Evidence from large population‐based studies for these numbers may be delayed in diagnosis.^2^, ^3^ Although approximately 10 percent of lung cancers in asymptomatic patients are detected on chest radiographs, several studies have suggested that most cancer patients are diagnosed after receiving regular check‐ups with the onset of cancer symptoms.^4^, ^5^, ^6^, ^7^ Persistent cough and dyspnea were reported as indicators of lung cancer from studies on the diagnosis of lung cancer.^8^, ^9^ In addition, patients with lung cancer commonly experience multiple symptoms for many months before seeking health care, including nonspecific systemic symptoms of loss of weight, loss of appetite and fatigue, or direct symptoms and physical signs caused by the primary tumor or intrathoracic or extrathoracic spread such as cough, chest pain, hemoptysis, Horner syndrome, enlarged lymph nodes, subcutaneous nodules, and facial swelling.^10^, ^11^, ^12^ However, low awareness of cancer symptoms may contribute to the delay in seeing a doctor, which leads to the diagnosis of late‐stage disease and poor cancer survival outcomes.^2^, ^13^ There was a better 5‐year‐survival rate for asymptomatic patients (18%) than for those with symptoms caused by the primary tumor (12%).^12^ Additionally, those with nonspecific symptoms had a 5‐year‐survival rate of 6%, and those with symptoms indicating metastatic disease had the worst survival rate, with none alive at 5 years. However, data from the national campaign in England showed that there was a 3.1% increase (*P* < 0.001) in the proportion of nonsmall cell lung cancer (NSCLC) diagnosed at stage I and a 2.3% increase (*P* < 0.001) in resections for NSCLC patients after raising public awareness about lung cancer symptoms.^14^ The results suggest that a sustained increase in resection surgeries may improve long‐term survival, especially in combination with raising public awareness of cancer symptoms and encouraging people with symptoms to visit the hospital. Although, some studies have shown that the majority of patients with advanced lung cancer experience some symptoms, including lung‐specific (cough, dyspnea, and chest pain) and systemic (appetite loss and fatigue) symptoms.^10^, ^11^, ^15^, ^16^ Some of the above symptoms, such as loss of appetite, weight loss, fatigue, fever or flu presentations, increase the risk of a lung cancer diagnosis.^17^ However, whether lung cancer symptoms are independently associated with the diagnosis of lung cancer is unknown, especially in terms of the pathological type and stage of the disease at diagnosis.^18^

The present study aimed to examine the symptoms and physical signs of lung cancer patients and determine whether these lung cancer symptoms and physical signs were independently associated with diagnosis; this was a hospital‐based multicenter 10‐year retrospective clinical epidemiological study from 2005 to 2014 focusing on primary lung cancer in mainland China. NSCLC is a major type of lung cancer and accounts for approximately 85% of all lung cancers.^19^ Therefore, this study focused on NSCLC.

## 2 MATERIALS AND METHODS

### 2.1 Study design and study sites

We conducted a hospital‐based multicenter 10‐year retrospective study of randomly selected pathology or cytology‐confirmed primary lung cancer cases from 2005 to 2014 via a medical chart review. The study was part of the Cancer Screening Program in Urban China (CanSPUC), which was supported by the central government.

The details of the hospital selection and case sampling methods have been described previously.^20^, ^21^ To ensure that our sample was geographically representative of China, we included eight tertiary hospitals from seven regions, including the North, Northeast, Central, South, East, Northwest, and Southwest, based on the traditional administrative district definitions by the National Bureau of Statistics using convenience sampling; the hospitals were the Shanxi Provincial Cancer Hospital (in Shanxi Province, North China), Liaoning Cancer Hospital (in Liaoning Province, Northeast China), Zhejiang Cancer Hospital (in Zhejiang Province, East China), Anhui Cancer Hospital (in Anhui Province, East China), Hunan Cancer Hospital (in Hunan Province, Central China), Guangxi Cancer Hospital (in Guangxi Autonomous Region, South China), Yunnan Cancer Hospital (in Yunnan Province, Southwest China), and Gansu Provincial Hospital (in Gansu Province, Northwest China).^20^, ^21^ The selected hospitals provided the medical records of patients with lung cancer diagnosed from 2005 to 2014. In addition, we excluded January and February to decrease any confounding effects for the Spring Festival holiday. During this period, most patients celebrate the Spring Festival and go home to spend the holiday with their families, so there were a small number of patients during this time period, which might be a potential confounding outcomes comparison.

### 2.2 Data collection

The details of the designed case report form (CRF) and data collection process have also been described previously.^18^, ^19^ The CRF was designed into an initial questionnaire by experts, and the CRF was revised repeatedly into a formal final questionnaire through several rounds of discussion and presurveys. The CRF was used to collect information on the enrolled patients, including the general information, demographic characteristics, symptoms, physical signs, the Eastern Cooperative Oncology Group (ECOG) performance scale (PS), pathological type, and stage determined by the American Joint Commission on Cancer (AJCC) seventh edition stage for lung cancer). All data were abstracted manually or exported by batch from the medical record system. To improve the data quality, we conducted three levels of organized protocol trainings including the national, provincial, and hospital level for local interviewers, who were clinical doctors or postgraduates with well‐trained health professionals over a period of 2 years in lung cancer, or staffs from department of medical record or cancer prevention with requirements of an educational background of clinical medicine or public health in lung cancer. In the process of data collection, the interviewers firstly screened the medical record in local hospitals and recorded whether to include one case according to the above inclusion criteria into a designed form (including age at diagnosis, gender, date at diagnosis, diagnostic information [pathological type and clinical stage], and other essential information). The criteria for excluded cases were indicated and an identity number was assigned for each eligible case, then completed information was extracted. All the variables were double‐entered into computer‐based database (EpiData 3.1) for consistency by two local well‐trained clerks, and then were sent to National Program Office for the missing value confirmation and logic check using coded programs in the SAS software. After several rounds of the above procedure, confirmed data were included for analysis. The following symptoms and physical signs were present before the cancer diagnosis. The symptoms included pain in the waist/shoulder/back, chronic cough, sputum with blood, shortness of breath, chest pain, dyspnea, recurrent bronchitis/pneumonia, hoarseness, amyosthenia, hyponatremia, metastases pain, headache, dizzy, sudden dyskinesia, weight loss, fatigue, fever, and others. The physical signs included neck and supraclavicular lymphadenectasis, lymphadenectasis in other locations, subcutaneous nodules, Horner syndrome, facial swelling, abnormal breath sounds, and others. All information, including age, sex, family history of lung cancer, body mass index (BMI), smoking history, alcohol consumption status, socioeconomic status, education level, symptoms, physical signs, ECOG PS, pathological type of the disease, and disease stage, were collected during the review of the medical charts. This study focused on NSCLC patients, and patients without available information on symptoms, physical signs, pathological type, and stage were excluded.

### 2.3 Data processing

According to the differences in BMI across the Chinese population, the categories in our study were classified by BMI according to the Chinese adult BMI classification made by the China Obesity Task Force.^22^ Namely, patients with BMIs <18.5 kg/m^2^ were low weight, 18.5‐23.9 kg/m^2^ were normal weight, 24.0‐27.9 kg/m^2 ^were overweight, and BMI ≥28.0 kg/m^2^ were obese in the present study. According to several economic indictors,^20^ the Anhui Province, Yunnan Province, Gansu Province, and Guangxi Autonomous Region were divided into the less‐developed region. The Liaoning, Zhejiang, Hunan, and Shanxi Provinces were divided into the well‐developed region to represent Northeast, North, South, and East China. According to the differences in education level, patients with a primary school education or below were defined as low education, those with a middle school and high school education were defined as middle education, and those with a college degree or above were defined as high education.

### 2.4 Statistical analysis

The baseline characteristics are described as frequencies and percentages for categorical variables and as means and standard deviations for continuous variables. Differences in categorical data between patients with and without symptoms and physical signs were compared with the chi‐square test or Fisher's exact test. Odds ratios (ORs) and 95% confidence intervals (CIs) were calculated using multivariate unconditional logistic regressions to describe associations between the symptoms and physical signs of lung cancer and age, sex, smoking history, drinking history, BMI, socioeconomic status, education level, disease stage at diagnosis, and pathological type of disease. For all analyses, the statistical software SAS version 9.4 was used. A *P* value < 0.05 was considered significant.

## 3 RESULTS

### 3.1 Patient characteristics

A total of 7184 patients diagnosed with lung cancer with disease stage information were surveyed between 2005 and 2014, and a total of 6398 NSCLC patients with available information about their symptoms and physical signs were included in this analysis. Table 1 describes the differences in the demographics of the patients with and without symptoms and physical signs. In our study, 4623 (72.3%) participants were men, and 1775 (27.7%) were women. The mean age for the overall population was 58.4 ± 10.1 years. We found that subjects who had symptoms were more likely to be younger (<65 years [71.4%] and ≥ 65 years [28.6%]), to be men (72.3%), and to have been diagnosed with stage III (34.4%) or IV (28.7%) disease than those who did not have symptoms. Additionally, the data suggested that the patients with symptoms were also more likely to have a normal BMI (55.2%), to be smokers (42.6%), to be not consume alcohol (67.3%), and to have a family history of lung cancer (91.6%) than the other populations. As seen in Table 1, we found that there were significant differences between patients with and without symptoms in relation to age (*P* = 0.0042), sex (*P* < 0.0001), smoking history (*P* < 0.0001), drinking history (*P* < 0.0001), BMI (*P* < 0.0001), socioeconomic status (*P* = 0.0003), education level (*P* < 0.0001), pathological type (*P* < 0.0001), and disease stage (*P* < 0.0001). Significant differences between patients with and without physical signs were also observed in relation to age (*P* = 0.0050), smoking history (*P* < 0.0001), drinking history (*P* < 0.0001), BMI (*P* = 0.0030), socioeconomic status (*P* < 0.0001), education level (*P* < 0.0001), pathological type (*P* < 0.0001), and disease stage (*P* < 0.0001).

**Table 1:** Demographic characteristics of all included NSCLC patients

Characteristics		Symptoms				Physical signs			
	Total,n (%)	No, n (%)	Yes, n (%)	*χ* ^2^	*P* value	No, n (%)	Yes, n (%)	*χ* ^2^	*P* value
Age at diagnosis									
＜45	609 (9.5)	100 (9.4)	509 (9.6)	13.21	0.0042	502 (9.0)	107 (12.7)	12.83	0.0050
45‐64	3963 (61.9)	616 (58.0)	3347 (62.7)			3470 (62.5)	493 (58.5)		
65‐70	912 (14.3)	161 (15.1)	751 (14.1)			797 (14.3)	115 (13.7)		
＞70	914 (14.3)	186 (17.5)	728 (13.6)			787 (14.2)	127 (15.1)		
Family history of lung cancer									
No	535 (8.3)	96 (9.0)	439 (8.2)	3.99	0.1361	461 (8.3)	74 (8.8)	0.83	0.6599
Yes	5859 (91.6)	965 (90.8)	4894 (91.7)			5091 (91.6)	768 (91.2)		
Unknown	4 (0.1)	2 (0.2)	2 (0.1)			4 (0.1)	0		
Body mass index (kg/m^2^)									
＜18.5	538 (8.4)	60 (5.6)	478 (9.0)	46.17	＜0.0001	457 (8.2)	81 (9.6)	16.04	0.0030
18.5‐24.0	3531 (55.2)	559 (52.6)	2972 (55.7)			3067 (55.2)	464（55.1）		
24.0‐28.0	1345 (21.0)	283 (26.6)	1062 (19.9)			1191 (21.4)	154 (18.3)		
≥28.0	240 (3.8)	58 (5.5)	182 (3.4)			219 (4.0)	21 (2.5)		
Unknown	744 (11.6)	103 (9.7)	641 (12.0)			622 (11.2)	122 (14.5)		
Smoking history									
Current	2728 (42.6)	367 (34.5)	2361 (44.2)	68.24	＜0.0001	2430 (43.7)	298 (35.4)	24.72	＜0.0001
Previous	839 (13.1)	104 (9.8)	735 (13.8)			711 (12.8)	128 (15.2)		
Never	2751 (43.0)	576 (54.2)	2175 (40.8)			2341 (42.2)	410 (48.7)		
Unknown	80 (1.3)	16 (1.5)	64 (1.2)			74 (1.3)	6 (0.7)		
Alcohol consumption status									
Yes	1763 (27.6)	230 (21.6)	1533 (28.7)	24.20	＜0.0001	1533 (27.6)	230 (27.3)	39.82	＜0.0001
No	4308 (67.3)	783 (73.7)	3525 (66.1)			3702 (66.6)	606 (72.0)		
Unknown	327 (5.1)	50 (4.7)	277 (5.2)			321 (5.8)	6 (0.7)		
Sex									
Male	4623 (72.3)	689 (64.8)	3934 (73.7)	35.20	＜0.0001	4030 (72.5)	593 (70.4)	1.62	0.2033
Female	1775 (27.7)	374 (35.2)	1401 (26.3)			1526 (27.5)	249 (29.6)		
Socioeconomic status									
Less	3223 (50.4)	589 (55.4)	2634 (49.4)	12.92	0.0003	3022 (54.4)	201 (23.9)	272.45	＜0.0001
Well	3175 (49.6)	474 (44.6)	2701 (50.6)			2534 (45.6)	641 (76.1)		
Education level									
Primary school or below	1696 (26.5)	225 (21.2)	1471 (27.6)	68.11	＜0.0001	1437 (25.9)	259 (30.8)	9.94	0.0190
Middle or high school	1765 (27.6)	294 (27.6)	1471 (27.5)			1544 (27.8)	221 (26.2)		
College degree or above	390 (6.1)	119 (11.2)	271 (5.1)			336 (6.0)	54 (6.4)		
Unknown	2547 (39.8)	425 (40.0)	2122 (39.8)			2239 (40.3)	308 (36.6)		
Pathological type									
Adenocarcinoma	2880 (45.0)	682 (64.1)	2198 (41.2)	192.35	＜0.0001	2493 (44.9)	387 (46.0)	20.98	＜0.0001
Squamous carcinoma	2991 (46.8)	309 (29.1)	2682 (50.3)			2449 (44.1)	321 (38.1)		
Others	527 (8.2)	72 (6.8)	455 (8.5)			614 (11.0)	134 (15.9)		
Stage									
I	1272 (19.9)	415 (39.0)	857 (16.1)	325.41	＜0.0001	1239 (22.3)	33 (3.9)	480.46	＜0.0001
IIA	532 (8.3)	95 (8.9)	437 (8.2)			507 (9.1)	25 (3.0)		
IIB	559 (8.7)	94 (8.9)	465 (8.7)			517 (9.3)	42 (5.0)		
IIIA	1334 (20.9)	186 (17.5)	1148 (21.5)			1232 (22.2)	102 (12.1)		
IIIB	862 (13.5)	87 (8.2)	775 (14.5)			683 (12.3)	179 (21.3)		
IV	1839 (28.7)	186 (17.5)	1653 (31.0)			1378 (24.8)	461 (54.7)		

Abbreviation: ECOG PS, Eastern Cooperative Oncology Group performance scale.

### 3.2 The outcomes of patients with symptoms and physical signs of lung cancer

**Figure 1:**
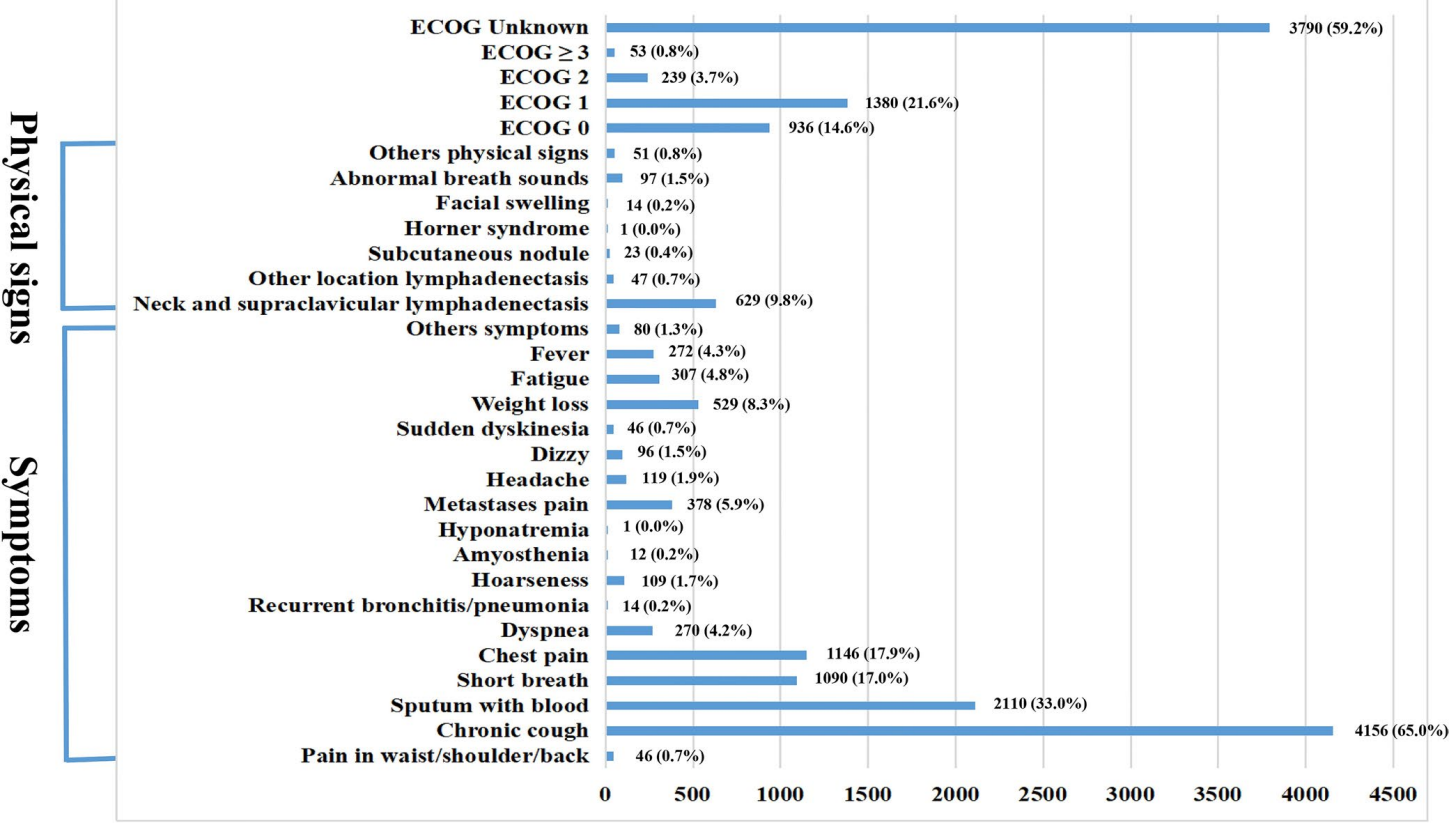
The outcomes of symptoms and physical signs

**Figure 2:**
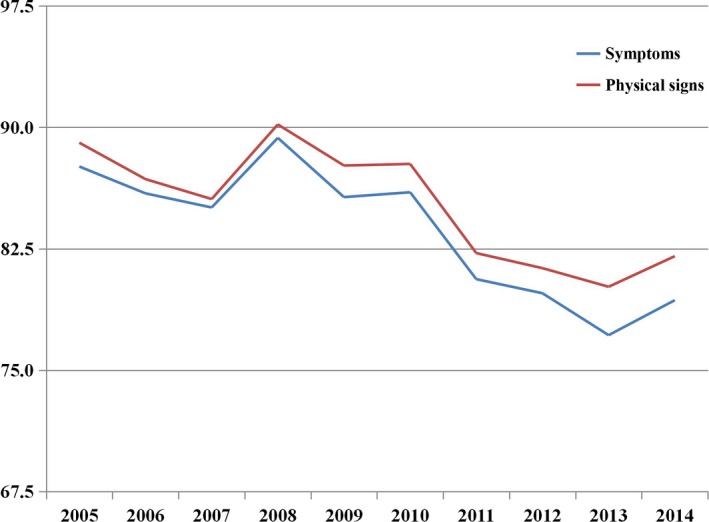
The incidences of symptoms and physical signs analyses in 10 years (2005‐2014)

The outcomes of patients with symptoms and physical signs of lung cancer are listed in Figure 1. The most common initial symptom and physical sign was chronic cough (4156, 65.0%), followed by sputum with blood (2110, 33.0%), chest pain (1146, 17.9%), shortness of breath (1090, 17.0%), neck and supraclavicular lymphadenectasis (629, 9.8%), weight loss (529, 8.3%), metastases pain (378, 5.9%), fatigue (307, 4.8%), fever (272, 4.3%), dyspnea (270, 4.2%), headache (119, 1.9%), and hoarseness (109, 1.7%). Additionally, patients with an ECOG PS of 1 accounted for 21.6% (1380) of the population, those with an ECOG PS of 0 accounted for 14.6% (936 patients) of the population, and the ECOG PS of 59.2% of the patients was unknown. We observed that most patients did not experience any symptoms and physical signs, and the proportion of symptoms seemed to be higher than the proportion of physical signs. The incidence of the symptoms and physical signs of lung cancer were analyzed for the years 2005‐2014, which yielded a line chart, as seen in Figure 2. The Figure 2 shows that the incidence of the symptoms and physical signs of lung cancer presented a downward tendency year by year.

### 3.3 Association between the selected risk factors and the symptoms and physical signs of NSCLC in China

The results of the analysis on the relationship between risk factors and the symptoms and physical signs of lung cancer are listed in Table 2. In multivariable logistic analyses, having a low body weight (OR = 0.667 [95% CI, 0.503‐0.886] for 18.5‐23.9 kg/m^2^, OR = 0.471 [95% CI, 0.349‐0.635] for 24.0‐27.9 kg/m^2^, and OR = 0.394 [95% CI, 0.264‐0.587] for more than 28.0 kg/m^2^, compared with 18.5 kg/m^2^, respectively), being a smoker (OR = 0.587 [95% CI, 0.509‐0.677] for nonsmokers compared with smokers), being a drinker (OR = 0.676 [95% CI, 0.576‐0.792] for nondrinkers compared with drinkers), being male (OR = 0.656 [95% CI, 0.570‐0.754] for female compared with male), having a low educational level (OR = 0.765 [95% CI, 0.634‐0.924] for middle or high school and OR = 0.348 [95% CI, 0.269‐0.450] for college degree or above, compared with primary school or below, respectively), and being from more developed regions (OR = 1.274 [95% CI, 1.116‐1.454] for well‐developed regions compared with less‐developed regions) was significantly associated with increased the probability of presenting symptoms. Being young (OR = 0.667 [95% CI, 0.530‐0.838] for patients with 45‐64 years old and OR = 0.677 [95% CI, 0.509‐0.901] for patients with 65‐70 years old compared with patients with less than 45 years old, respectively), being overweight (OR = 0.730 [95% CI, 0.546‐0.975] for 24.0 ~ 27.9 kg/m^2^ and OR = 0.541 [95% CI, 0.326‐0.898] for more than 28.0 kg/m^2^, compared with 18.5 kg/m^2^, respectively), being a smoker (OR = 1.428 [95% CI, 1.217‐1.675] for nonsmokers compared with smokers) and being from well‐developed regions (OR = 3.803 [95% CI, 3.218‐4.494] for well‐developed regions compared with less‐developed regions) were associated with a high probability that the participant would develop physical signs. Compared with patients with adenocarcinoma, patients with squamous carcinoma had a higher probability of developing symptoms (OR = 2.885, 95% CI 2.477‐3.359) but not physical signs (OR = 0.844, 95% CI 0.721‐0.989). In addition, patients with stage IIA, stage IIB, stage IIIA, stage IIIB, and stage IV disease were 2.228 (95% CI, 1.733‐2.863), 2.396 (95% CI, 1.864‐3.078), 2.989 (95% CI, 2.461‐3.630), 4.314 (95% CI, 3.357‐5.543), and 4.304 (95% CI, 3.553‐5.213) times more likely to develop symptoms than those with stage I disease, respectively, and 1.851 (95% CI, 1.090‐3.144), 3.049 (95% CI, 1.911‐4.866), 3.108 (95% CI, 2.082‐4.637), 9.837 (95% CI, 6.708‐14.425), and 12.557 (95% CI, 8.749‐18.023) times more likely to develop physical signs than those with stage I disease, respectively. The odds of developing both symptoms and physical signs were higher in patients with advanced‐stage disease than in those with early‐stage disease (*P* < 0.0001).

**Table 2:** The influencing factors of symptoms and physical signs in the multivariate unconditional logistic regressions

	Symptoms			Physical signs		
	OR	95% CI	*P* value	OR	95% CI	*P* value
Age at diagnosis						
＜45	Reference					
45‐64	1.067	0.847‐1.345	0.5795	0.667	0.530‐0.838	0.0005
65‐70	0.916	0.697‐1.205	0.5320	0.677	0.509‐0.901	0.0075
＞70	0.769	0.588‐1.005	0.0547	0.757	0.572‐1.002	0.0519
Body mass index (kg/m2)						
＜18.5	Reference					
18.5 ≤ BMI<24.0	0.667	0.503‐0.886	0.0051	0.854	0.661‐1.102	0.2248
24.0 ≤ BMI<27.9	0.471	0.349‐0.635	＜0.0001	0.730	0.546‐0.975	0.0310
≥28.0	0.394	0.264‐0.587	＜0.0001	0.541	0.326‐0.898	0.0174
Unknown	0.781	0.556‐1.097	0.1541	1.107	0.815‐1.502	0.5161
Smoking history						
Yes	Reference					
Previous	1.099	0.870‐1.387	0.4290	1.468	1.174‐1.835	0.0008
Never	0.587	0.509‐0.677	＜0.0001	1.428	1.217‐1.675	＜0.0001
Unknown	0.622	0.356‐1.087	0.0955	0.661	0.285‐1.532	0.3347
Alcohol consumption status						
Yes	Reference					
No	0.676	0.576‐0.792	＜0.0001	1.091	0.927‐1.284	0.2948
Unknown	0.831	0.597‐1.158	0.2749	0.125	0.055‐0.283	＜0.0001
Sex						
Male	Reference					
Female	0.656	0.570‐0.754	＜0.0001	1.109	0.946‐1.300	0.2034
Socioeconomic status						
Less	Reference					
Well	1.274	1.116‐1.454	0.0003	3.803	3.218‐4.494	＜0.0001
Education level						
Primary school or below	Reference					
Middle or high school	0.765	0.634‐0.924	0.0053	0.794	0.655‐0.963	0.0194
College degree or above	0.348	0.269‐0.450	＜0.0001	0.892	0.650‐1.223	0.4774
Unknown	0.764	0.641‐0.910	0.0025	0.763	0.639‐0.912	0.0020
Pathological type						
Adenocarcinoma	Reference					
Squamous carcinoma	2.885	2.477‐3.359	＜0.0001	0.844	0.721‐0.989	0.0360
Others	1.762	1.416‐2.192	＜0.0001	1.406	1.133‐1.744	0.0019
Stage						
I	Reference					
IIA	2.228	1.733‐2.863	＜0.0001	1.851	1.090‐3.144	＜0.0001
IIB	2.396	1.864‐3.078	＜0.0001	3.049	1.911‐4.866	＜0.0001
IIIA	2.989	2.461‐3.630	＜0.0001	3.108	2.082‐4.637	＜0.0001
IIIB	4.314	3.357‐5.543	＜0.0001	9.837	6.708‐14.425	＜0.0001
IV	4.304	3.553‐5.213	＜0.0001	12.557	8.749‐18.023	＜0.0001

Abbreviations:CI, confidence interval; OR, odds ratios.

### 3.4 Symptoms and physical signs of lung cancer in the distribution of risk factors for NSCLC in China

The detailed symptoms and physical signs of lung cancer in the distribution of pathological type and disease stage are listed in Tables 3 and 4, respectively. The data showed that patients with chronic cough (*P* < 0.0001), sputum with blood (*P* < 0.0001), hoarseness (*P* = 0.0071), weight loss (*P* = 0.0104), fatigue (*P* = 0.0243), and fever (*P* = 0.0007) were more likely to have squamous carcinoma than nonsquamous carcinoma. While the symptoms of metastases pain (*P* < 0.0001), dizziness (*P* = 0.0472), and neck and supraclavicular lymphadenectasis (*P* = 0.0006) were more likely to occur in patients with nonsquamous carcinoma than patients with other carcinomas. Additionally, patients with chronic cough were significantly more likely to have stage III disease (*P* < 0.0001) than diseases of other stages. Patients with stage IV disease had a higher percentage of chest pain (*P* < 0.0001), shortness breath (*P* < 0.0001), dyspnea (*P* = 0.0003), metastases pain (*P* < 0.0001), weight loss (*P* < 0.0001), fatigue (*P* < 0.0001), neck and supraclavicular lymphadenectasis (*P* < 0.0001), lymphadenectasis in other locations (*P* < 0.0001), subcutaneous nodules (*P* < 0.0001), facial swelling (*P* = 0.0187), and abnormal breath sounds (*P* < 0.0001) than patients with disease of other stages. Additionally, patients with early‐stage disease had a better ECOG PS in than those with advanced‐stage disease.

**Table 3:** The distributions symptoms and physical signs in different pathological type

	Total	Squamous carcinoma	Adenocarcinoma	Others	*χ* ^2^	*P* value	Nonsquamous carcinoma	Squamous vs Nonsquamous* χ* ^2^	Squamous vs Nonsquamous* P* value
	N (%)	N (%)	N (%)	N (%)			N (%)		
Symptoms									
Pain in waist/shoulder/back	46 (0.7)	13 (0.5)	27 (0.9)	6 (0.8)	4.42	0.1098	33 (0.9)	4.27	0.0389
Chronic cough	4156 (65.0)	2100 (75.8)	1573 (54.6)	483 (64.6)	278.69	＜0.0001	2056 (56.7)	252.84	＜0.0001
Sputum with blood	2110 (33.0)	1208 (43.6)	673 (23.4)	229 (30.6)	263.89	＜0.0001	902 (24.9)	249.78	＜0.0001
Short breath	1090 (17.0)	449 (16.2)	512 (17.8)	129 (17.2)	2.48	0.2889	641 (17.7)	2.36	0.1241
Chest pain	1146 (17.9)	484 (17.5)	526 (18.3)	136 (18.2)	0.64	0.7251	662 (18.2)	0.64	0.4237
Dyspnea	270 (4.2)	134 (4.8)	111 (3.9)	25 (3.3)	4.99	0.0824	136 (3.7)	4.61	0.0318
Recurrent bronchitis/pneumonia	14 (0.2)	8 (0.3)	3 (0.1)	3 (0.4)	3.49	0.1744	6 (0.2)	1.10	0.2952
Hoarseness	109 (1.7)	61 (2.2)	31 (1.1)	17 (2.3)	12.32	0.0021	48 (1.3)	7.25	0.0071
Amyosthenia	12 (0.2)	5 (0.2)	4 (0.1)	3 (0.4)	2.19	0.3340	7 (0.2)	0.01	0.9093
Hyponatremia	1 (0.0)	0	1 (0.0)	0	1.22	0.5429	1 (0.0)	0.76	0.3822
Metastases pain	378 (5.9)	121 (4.4)	206 (7.2)	51 (6.8)	20.96	＜0.0001	257 (7.1)	20.84	＜0.0001
Headache	119 (1.9)	42 (1.5)	56 (1.9)	21 (2.8)	5.58	0.0613	77 (2.1)	3.16	0.0754
Dizzy	96 (1.5)	32 (1.2)	44 (1.5)	20 (2.7)	9.22	0.0100	64 (1.8)	3.94	0.0472
Sudden dyskinesia	46 (0.7)	17 (0.6)	22 (0.8)	7 (0.9)	1.00	0.6053	29 (0.8)	0.76	0.3839
Weight loss	529 (8.3)	257 (9.3)	203 (7.0)	69 (9.2)	10.27	0.0059	272 (7.5)	6.57	0.0104
Fatigue	307 (4.8)	152 (5.5)	118 (4.1)	37 (4.9)	6.01	0.0494	155 (4.3)	5.08	0.0243
Fever	272 (4.3)	145 (5.2)	97 (3.4)	30 (4.0)	12.21	0.0022	127 (3.5)	11.60	0.0007
Others	80 (1.3)	27 (1.0)	41 (1.4)	12 (1.6)	3.16	0.2056	53 (1.5)	3.01	0.0829
Physical signs									
Neck and supraclavicular lymphadenectasis	629 (9.8)	232 (8.4)	294 (10.2)	103 (13.8)	20.18	＜0.0001	397 (10.9)	11.68	0.0006
Other location lymphadenectasis	47 (0.7)	24 (0.9)	16 (0.6)	7 (0.9)	2.34	0.3101	23 (0.6)	1.16	0.2806
Subcutaneous nodule	23 (0.4)	8 (0.3)	8 (0.3)	7 (0.9)	7.86	0.0196	15 (0.4)	0.68	0.4092
Horner syndrome	1 (0.0)	0	1 (0.0)	0	1.22	0.5429	1 (0.03)	0.76	0.3822
Facial swelling	14 (0.2)	9 (0.3)	1 (0.0)	4 (0.5)	9.32	0.0095	5 (0.1)	2.52	0.1125
Abnormal breath sounds	97 (1.5)	41 (1.5)	43 (1.5)	13 (1.7)	0.28	0.8690	56 (1.5)	0.04	0.8371
Others	51 (0.8)	16 (0.6)	30 (1.0)	5 (0.7)	4.02	0.1338	35(1.0)	2.98	0.0845
ECOG PS									
0	936 (14.6)	372 (13.4)	477 (165)	87 (11.7)	23.86	0.0024	564 (15.6)	12.21	0.0159
1	1380 (21.6)	600 (21.7)	613 (21.3)	167 (22.3)			780 (21.5)		
2	239 (3.7)	123 (4.5)	92 (3.2)	24 (3.2)			116 (3.2)		
≥3	53 (0.8)	20 (0.7)	26 (0.9)	7 (0.9)			33 (0.9)		
Unknown	3790 (59.2)	1655 (59.7)	1672 (58.1)	463 (61.9)			2135 (58.8)		

Abbreviation: ECOG PS, Eastern Cooperative Oncology Group performance scale.

**Table 4:** The distributions symptoms and physical signs in different stages

	Stage I	Stage II	Stage III	Stage IV	*χ* ^2^	*P* value	Stage I‐III	Stage I‐III vs IV *χ* ^2^	I‐III vs IV* P* value
	N (%)	N (%)	N (%)	N (%)			N (%)		
Symptoms									
Pain in waist/shoulder/back	6 (0.5)	7 (0.6)	9 (0.4)	24 (1.3)	12.97	0.0047	22 (0.5)	12.42	0.0004
Chronic cough	652 (51.3)	731 (67.0)	1599 (72.8)	1174 (63.8)	167.44	＜0.0001	2982 (65.4)	1.42	0.2336
Sputum with blood	360 (28.3)	410 (37.6)	844 (38.4)	496 (27.0)	82.63	＜0.0001	1614 (35.4)	42.15	＜0.0001
Short breath	133 (10.5)	149 (13.7)	376 (17.1)	432 (23.5)	102	＜0.0001	658 (14.4)	76.07	＜0.0001
Chest pain	147 (11.6)	162 (14.8)	375 (17.1)	462 (25.1)	107.97	＜0.0001	684 (15)	91.26	＜0.0001
Dyspnea	25 (2.0)	29 (2.7)	112 (5.1)	104 (5.7)	36.16	＜0.0001	166 (3.6)	13.15	0.0003
Recurrent bronchitis/pneumonia	0	3 (0.3)	6 (0.3)	5 (0.3)	3.48	0.3231	9 (0.2)	0.33	0.5640
Hoarseness	3 (0.2)	6 (0.5)	46 (2.1)	54 (2.9)	43.73	＜0.0001	55 (1.2)	23.42	＜0.0001
Amyosthenia	0	2 (0.2)	3 (0.1)	7 (0.4)	6.36	0.0954	5 (0.1)	5.14	0.0234
Hyponatremia	0	0	0	1 (0.1)	2.48	0.4790	0	2.48	0.1153
Metastases pain	13 (1)	37 (3.4)	91 (4.1)	237 (12.9)	240.5	＜0.0001	141 (3.1)	226.14	＜0.0001
Headache	4 (0.3)	6 (0.5)	19 (0.9)	90 (4.9)	131.55	＜0.0001	29 (0.6)	130.15	＜0.0001
Dizzy	10 (0.8)	3 (0.3)	19 (0.9)	64 (3.5)	70.24	＜0.0001	32 (0.7)	68.44	＜0.0001
Sudden dyskinesia	1 (0.1)	1 (0.1)	2 (0.1)	42 (2.3)	88.54	＜0.0001	4 (0.1)	88.54	＜0.0001
Weight loss	28 (2.2)	61 (5.6)	181 (8.2)	259 (14.1)	154.04	＜0.0001	270 (5.9)	115.08	＜0.0001
Fatigue	27 (2.1)	30 (2.7)	91 (4.1)	159 (8.6)	91.62	＜0.0001	148 (3.2)	83.64	＜0.0001
Fever	39 (3.1)	44 (4)	114 (5.2)	75 (4.1)	9.42	0.0242	197 (4.3)	0.19	0.6631
Others	14 (1.1)	15 (1.4)	19 (0.9)	32 (1.7）	6.58	0.0866	48 (1.1)	5.01	0.0252
Physical signs									
Neck and supraclavicular lymphadenectasis	15 (1.2)	43 (3.9)	220 (10.0)	351 (19.1)	327.9	＜0.0001	278 (6.1)	249.39	＜0.0001
Other location lymphadenectasis	0	2 (0.2)	15 (0.7)	30 (1.6)	34.32	＜0.0001	17 (0.4)	28.46	＜0.0001
Subcutaneous nodule	2 (0.2)	1 (0.1)	1 (0.05)	19 (1.0)	32.98	＜0.0001	4 (0.1)	32.70	＜0.0001
Horner syndrome	0	0	0	1 (0.1)	2.48	0.4790	0	2.48	0.1153
Facial swelling	0	1 (0.1)	5 (0.2)	8 (0.4)	7.54	0.0565	6 (0.1)	5.53	0.0187
Abnormal breath sounds	8 (0.6)	13 (1.2)	26 (1.2)	50 (2.7)	26.91	＜0.0001	47 (1.0)	25.01	＜0.0001
Others	8 (0.6)	7 (0.6)	20 (0.9)	16 (0.9)	1.27	0.7361	35 (0.8)	0.17	0.6770
ECOG PS									
0	284 (22.3)	236 (21.6)	349 (15.9)	67 (3.7)	536.48	＜0.0001	869 (19.0)	441.38	＜0.0001
1	190 (14.9)	185 (17.0)	540 (24.6)	465 (25.3)			915 (20.1)		
2	6 (0.5)	10 (0.9)	66 (3.0)	157 (8.5)			82 (1.8)		
≥3	1 (0.1)	2 (0.2)	11 (0.5)	39 (2.1)			14 (0.3)		
Unknown	791 (62.2)	658 (60.3)	1230 (56.0)	1111 (60.4)			2679 (58.8)		

Abbreviation: ECOG PS, Eastern Cooperative Oncology Group performance scale.

## 4 DISCUSSION

In this hospital‐based multicenter 10 years (2005 to 2014) retrospective clinical epidemiological study for primary lung cancer in mainland China, 6398 NSCLC patients were included to explicitly provide quantitative information about their symptoms and physical signs; this study included Chinese NSCLC patients to determine whether any relationships existed between the pathological type and disease stage the presence of lung cancer symptoms.

This analysis showed that the most common symptoms and physical signs of lung cancer were chronic cough (65.0%), sputum with blood (33.0%), chest pain (17.9%), shortness of breath (17.0%), neck and supraclavicular lymphadenectasis (9.8%), weight loss (8.3%), metastases pain (5.9%), fatigue (4.8%), fever (4.3%), and dyspnea (4.2%). Early studies reported that 60%‐70% of the patients ultimately diagnosed with pulmonary cancer presented with one or more local symptoms (cough, dyspnea, or chest pain), and 41% had general symptoms (fever, weight loss, or tiredness).^10^ An English study reported similar results that the most common symptoms were cough (56.2%), fatigue or tiredness (45.1%), breathlessness (41.2%), chest/shoulder pain (35.3%), and coughing up blood (21.6%).^23^ Although the incidence of general symptoms was relatively low in our study, we found that, in keeping with the above studies, the local symptoms of chronic cough, sputum with blood, and chest pain were the main symptoms for lung cancer patients. These findings indicated that the above symptoms might be signs of lung cancer.

An important advantage of this study is the information on the relationships between the symptoms and physical signs of lung cancer and the pathological type and disease stage of NSCLC. A previous study suggested that some symptoms, such as loss of appetite, weight loss, fatigue, and fever/flu presentations, were associated with a high‐lung cancer risk.^17^ Furthermore, some of the presented symptoms, such as jaundice, are likely to represent advanced colorectal cancer.^24^ A study based on the general population indicated that the incidences of these symptoms were not associated with disease stage.^25^ However, few studies have reported the relationship between the symptoms and physical signs of lung cancer and the stage of cancer, particularly for lung cancer. In our study, the incidence of symptoms and physical signs was higher in patients with advanced‐stage disease than in those with early‐stage disease; the odds of patients with stage IIA, stage IIB, stage IIIA, stage IIIB, and stage IV disease presenting symptoms were 2.228 (95% CI, 1.733‐2.863), 2.396 (95% CI, 1.864‐3.078), 2.989 (95% CI, 2.461‐3.630), 4.314 (95% CI, 3.357‐5.543), and 4.304 (95% CI, 3.553‐5.213) times the odds of those with stage I disease, respectively, and the odds of these patients presenting physical signs were 1.851 (95% CI, 1.090‐3.144), 3.049 (95% CI, 1.911‐4.866), 3.108 (95% CI, 2.082‐4.637), 9.837 (95% CI, 6.708‐14.425), and 12.557 (95% CI, 8.749‐18.023) times the odds of those with stage I disease, respectively. These results suggested that there are significant associations between the symptoms and physical signs of lung cancer and disease stage; the more advanced the disease stage, the more likely that symptoms and physical signs are present. Patients were perhaps more likely to have advance‐stage disease if they did experience symptoms or physical signs. One explanation for these findings is that most patients with lung cancer are asymptomatic or the disease is left undetected in its early stage.^14^ Another explanation is that the low awareness of cancer symptoms may contribute to delays in seeing a doctor, which leads to the diagnoses of late‐stage disease. Thus, raising public awareness of the symptoms and physical signs of cancer and encouraging people with those indicators to visit hospitals is necessary to achieve favorable clinical outcomes. Few studies have reported whether associations exist between the symptoms and physical signs and the pathological characteristics of lung cancer. In our analysis, squamous carcinoma significantly increased the risk of developing symptoms, but not physical signs (OR = 0.844, 95% CI 0.721‐0.989) compared with adenocarcinoma (OR = 2.885, 95% CI 2.477‐3.359). Additionally, patients with squamous carcinoma were perhaps less likely to present symptoms of chronic cough, sputum with blood, hoarseness, and fever than patients with other carcinomas. This may be because squamous cell lung cancer is a common central disease, which leads to lung‐specific symptoms. We look forward to further prospective cohort studies to explore the results.

Notably, the incidence of the symptoms and physical signs of lung cancer presented a downward tendency year by year. Studies have verified that surgical resection remains a major intervention that can improve long‐term survival for patients with early‐stage lung cancer,^23^, ^26^ and evidence has indicated that high resection rates were associated with a reduced risk of death in a population‐based study.^27^ There was a 3.1% increase (*P* < 0.001) in the proportion of NSCLC diagnosed at stage I and a 2.3% increase (*P* < 0.001) in the number of resections for NSCLC after raising public awareness of lung cancer symptoms in an English study.^14^ Our results indicate that a higher number of people were diagnosed with NSCLC after undergoing a health examination than only relying on the onset of symptoms year by year. Unfortunately, most patients were still diagnosed at relatively advanced stages (stage III or IV), and nearly 30% of patients were stage IV.

In the previous findings reported in other studies,^25^, ^28^ women generally reported more symptoms than men. However, in our study, we observed that men were more likely to report symptoms than women. The reason for this result might be that more than 70% of the participants were male, which led to an opposite result. Moreover, in multivariable logistic analyses, having a low body weight, being a smoker and being a drinker were significantly associated with a high probability of developing symptoms. Being young, having a low body weight and being a smoker were associated with a high probability that the participants had physical signs.

In this study, a low education level was found to be associated with a high incidence of the symptoms and physical signs of lung cancer. Patients with advanced‐stage disease were more likely to experience symptoms and physical signs than patients with early‐stage disease. There is evidence that a low educational level was associated with a high risk of being diagnosed with advanced stage lung cancer. Patients with a high education level were less likely to have stage IV lung cancer than those in the illiterate group (OR = 0.52, 0.63, 0.71, and 0.64 for the primary school, middle school, high school, college degree, or above subgroups, respectively).^20^ The findings suggest that patients in the most socioeconomically deprived areas might be correlated with a higher risk of advanced‐stage lung cancer, and a high educational level might be associated with a low risk of being diagnosed with advanced‐stage disease for both men and women. In addition, patients with a low educational level or low income were found to be less likely to seek medical advice or undergo treatment for the onset of symptoms than patients with a high education level or high income.^29^ One explanation is that patients with a low level of education also have a low awareness of the cancer symptoms, which leads to delays in seeing a doctor and contributes to the late stage at diagnosis. Another possible explanatory factor, which is in line with the report from India, is that financial constraint was reported as the main reason for not promptly seeking help, even for patients who suspected cancer.^30^ Interestingly, we found that patients from high‐income regions were likely to report symptoms, but not physical signs. Perhaps patients with high incomes have financial support and are thus likely to seek help promptly when they develop symptoms.

There are some limitations in our study that should be considered when interpreting our results. First, we used hospital‐based data, and the patients from the leading public cancer hospital of each province may not represent the entire population of that area. The data were collected with surveys rather than with random probability sampling, which could have made the sample less representative. Second, all included participants were diagnosed with lung cancer, and thus, there is no comparison between NSCLC patients and controls. Third, patients without available information about their symptoms, physical signs, pathological subtype, and disease stage were excluded from this analysis, which inevitably led to bias.

In summary, the symptoms and physical signs of lung cancer were associated with the pathological and clinical stage of NSCLC at diagnosis. Patients were perhaps likely to be diagnosed with advanced‐stage disease when they experienced symptoms or physical signs, and patients with squamous carcinoma were more likely to develop symptoms, but not physical signs, than patients with adenocarcinoma. Further prospective cohort studies are needed to explore these results.

## CONFLICT OF INTEREST

The authors declare no financial disclosures or conflict of interest.
